# Editorial: An update on neurological disorders post COVID-19 infection

**DOI:** 10.3389/fneur.2023.1229843

**Published:** 2023-07-13

**Authors:** Beatrice Paradiso, Clara Limback, Tao Su, Weiping Liao, Anastasios Mpotsaris

**Affiliations:** ^1^Department of Biomedical, Surgical and Dental Sciences, Faculty of Medicine and Surgery, Lino Rossi Research Center, University of Milan, Milan, Italy; ^2^Anatomic Pathology Unit, Dolo Hospital Venice, Venice, Italy; ^3^Department of Neuropathology and Ocular Pathology, Oxford University Hospitals NHS Foundation Trust, Oxford, United Kingdom; ^4^Key Laboratory of Neurogenetics and Channelopathies of Guangdong Province and the Ministry of Education of China, The Second Affiliated Hospital, Institute of Neuroscience, Guangzhou Medical University, Guangzhou, China; ^5^München Hospital, Munich, Germany; ^6^Faculty of Medicine, University Hospital Magdeburg, Magdeburg, Germany

**Keywords:** SARS-CoV-2, long-COVID syndrome, post-acute sequelae of COVID-19 (PASC), neuro-PASC, neuroinvasion, immunity, blood-brain barrier (BBB), brainstem nuclei

A new zoonotic coronavirus epidemic began in December 2019 in the city of Wuhan, China, and has affected almost the entire world. The World Health Organization (WHO) named this coronavirus 2019-nCoV, and COVID-19 the disease caused by it. On 11 March 2020, WHO declared the COVID-19 outbreak a global pandemic (1).

Globally, as of 12 April 2023, WHO reported 762,791,152 confirmed cases of COVID-19, including 6,897,025 deaths. As of 11th April 2023, a total of 13,340,343,269 vaccine doses have been administered. Three million new cases and over 23,000 deaths were reported in the previous 28 days (13 March to 9 April 2023), a decrease of 28% and 30%, respectively, compared to the previous 28 days (13 February to 12 March 2023). However, in opposition to the overall trend, important increases in reported cases and deaths were observed in the South-East Asia and Eastern Mediterranean regions and in numerous other countries (2). The world is not yet at the end of the COVID-19 epidemic because new virus variants are expected. However, on 4^th^ March 2023, the head of WHO declared “with great hope” an end to COVID-19 as a global public health emergency, stressing that this does not mean that the disease is no longer a worldwide threat (3).

The severe acute respiratory syndrome coronavirus 2 (SARS-CoV-2) causes COVID-19, a form of atypical pneumonia with multiple organ dysfunction but also simple respiratory flu-like symptoms; infection can be prevented or attenuated by vaccination. As in most respiratory infections, including influenza, SARS-CoV-2 infection viral shedding reaches the highest level in the nasopharynx and the nasal cavity mucosa is one of the most relevant sites of viral activity. Spinato et al. suggest, in a preliminary study, good compliance and subjective satisfaction for nasal lavages with saline solution in patients with newly diagnosed SARS-CoV-2 infection. The treatment showed effectiveness in reducing nasal symptoms of SARS-CoV-2 infection, compared to the control group. Hence, the nasopharyngeal route of viral dissemination and the easy administration of nasal sprays explains the rationale of the intranasal vaccine models that are under investigation.

According to the systematic review by Purja et al., the neurological complications of COVID-19 are diverse, and direct viral neuroinvasion is rare. The authors identify 2,387 studies and include 167 studies in which SARS-CoV-2 CSF PCR assay was performed in 101 patients. The SARS-CoV-2 PCR assay was positive in only four CSF samples out of the 101 cases. Olfactory dysfunction was present in only two of these four cases. The central and peripheral neurological manifestations observed were heterogeneous. The most common neurological diagnoses were Guillain-Barré syndrome (GBS) and its variants (24%), followed by encephalopathy (21%).

SARS-CoV-2 infection is a global health challenge producing significant post-acute sequelae and 30% of COVID-19 patients reported persistent symptoms for up to 9 months after illness. Patients who recover from COVID-19 and experience symptoms that persist for a protracted period after the primary infection are defined as having long-COVID (4 weeks after the primary infection) or post-COVID syndrome (12 weeks after the primary infection) (Schulze et al.). These patients are given the diagnosis of long COVID, post-acute COVID-19 syndrome (PACS), or post-acute sequelae of COVID-19 (PASC). It remains unclear whether long-COVID is a different disease entity than COVID-19 with unclear pathophysiology or a spectrum of prolonged viral infection (4).

Long COVID is frequently accompanied by new-onset conditions, mainly cardiovascular, thrombotic, or cerebrovascular disease; type 2 diabetes; myalgic encephalomyelitis/chronic fatigue syndrome (ME/CFS); postural orthostatic tachycardia syndrome; and other dysautonomic events (Carmona-Torre et al.). There are no validated effective treatments yet and these disabling symptoms can last for years. In particular, ME/CFS and dysautonomia may be lifelong conditions.

There are several possible causes of long COVID. Several hypotheses regarding its pathogenesis have been considered, including the persistent reservoir of SARS-CoV-2 in tissues; immune dysregulation with or without reactivation of the primary pathogen; and herpesviruses such as Epstein–Barr virus (EBV) and human herpesvirus 6 (HHV-6). Furthermore, the impact of SARS-CoV-2 on the microbiota, and the virome in particular; autoimmunity and other dysregulation of the immune system; microclotting with endothelial dysfunction; alterations in brainstem signaling and/or vagus nerve; and genetic causes have been considered (5).

Neuro-PASC involves direct or indirect brain invasion by the virus. The virus can then cause brain dysfunction and neuronal damage through direct cytolysis or secondary inflammatory and immune responses (indirect effects).

Direct invasion is uncommon (6). The virus can infect the Peripheral Nervous System (PNS) or CNS by direct infection of nerve endings (including olfactory, trigeminal, optic, and vagus nerves) gaining access to the CNS via the transport machinery of nerves and ganglions (7). The indirect mechanism is more frequent and involves the infection of cells of the circulatory system which carry the infection through the blood-brain barrier (BBB) into the CNS.

There are three main mechanisms by which a virus may cross the BBB: transcellular migration, paracellular migration, and the “Trojan horse” strategy. During transcellular migration, viruses enter the host endothelial cells to cross the BBB. In paracellular migration, viruses invade tight junctions formed by the endothelial cells of the BBB. With the Trojan horse strategy, viral particles are phagocytized by neutrophils and macrophages (8). Recently, some viral specialized molecules, called fusogens, have been recognized that fuse the viral envelope with neuronal or glioneuronal cell membranes and enter cells producing syncytial units among neurons and glia. This still poorly characterized, difficult-to-detect event could explain some of the neurological consequences of viral infections of the nervous system (9).

Viral BBB crossing determines three principal pathogenetic events: endotheliopathy, inflammatory response, and immune activation. These trigger astrocyte and microglia activation, proinflammatory cytokine release (Mehboob et al.), and CNS-specific immune activation which may be responsible for neural tissue injury and neurological symptoms of neuro-Covid (10). The neurological sequelae of Covid infection are frequently immune-mediated (Vavougios et al.).

Although COVID-19 may affect the incidence of specific neurological diseases, it is still to be determined whether this differs from the risk following other respiratory viral and bacterial infections. Zarifkar et al., study the frequency of neurodegenerative, cerebrovascular, and immune-mediated neurological diseases in outpatients post-COVID-19 compared to healthy control individuals and those with other respiratory tract infections. The risk of specific neurodegenerative and cerebrovascular, but not neuroimmune, disorders was increased in individuals with previous COVID-19 compared to healthy controls. However, with the exception of ischemic stroke, most neurological disorders were not more frequent after COVID-19 than after Influenza A/B and bacterial pneumonia.

Respiratory distress in patients with acute Covid-19 or in those with post-COVID syndrome is not exclusively due to atypical pneumonia. Both Vecchio et al. and Jareonsettasin et al. in 2022 demonstrated that inappropriate ventilatory homeostatic responses in individuals with acute COVID-19 may be related to direct brainstem involvement with overlapping indirect inflammatory mechanisms (8, 10–12) acting on the peripheral nervous system (Figure 1 by Jareonsettasin et al.). Weich et al. analyze the symptom of motor fatigue in post-COVID syndrome. All the patients included in Weich's clinical trial were not initially hospitalized and at the beginning displayed mild symptoms. Although they presented absolute values of oxygen uptake and ventilation within the normal range, they manifested mild anomalies in ventilation and chronic fatigue. These symptoms were not caused by organic lesions of the central motor system. In this study, Weich et al. do not exclude potential organic causes for chronic fatigue in long Covid disease such as mitochondrial dysfunction, endothelial dysfunction, chronic inflammation, autoimmunity, dysregulation of specific cytokines, or psychiatric and psychosomatic comorbidities, but rule out involvement of the central motor nuclei and cardiac and pulmonary deficit. Furthermore, it is not clear whether the mild anomalies in ventilation were caused by metabolic or psychogenic alterations too. Additional investigations of the psychiatric disorders in post-COVID-19 syndrome are necessary to understand the frequent association observed by Lier et al. between long COVID disease and a particular subset of patients with predominant fatigue, somatization, and depression. These patients present minor or no post-COVID cardiopulmonary distress and prominent psychiatric manifestations.

**Figure 1:**
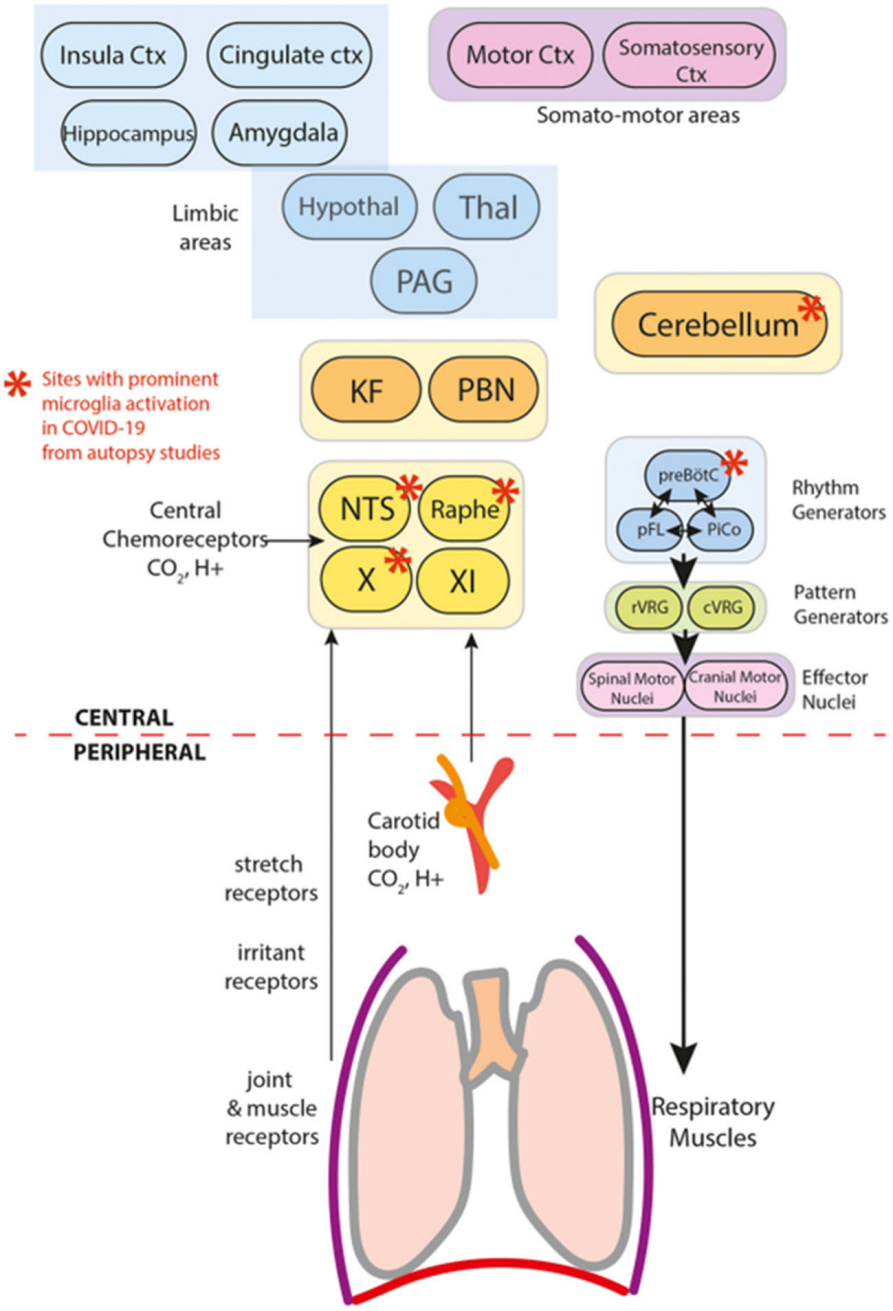
Components of breathing control in the context of COVID-19 (Jareonsettasin et al.).

COVID-19 is a complex syndrome and a complex of syndromes with early complications and late sequelae involving the brain, the brainstem, and the autonomic peripheral system, all of which are still poorly characterized.

For this reason, the Neuroinfectious Diseases section in Frontiers in Neurology “opens the door” to new “Frontiers” in scientific adventures (An Update on Neurological Disorders Post COVID-19 Infection Vol 2: cardiovascular effects, neuro-cardiac and neuro-respiratory autonomic dysfunctions).

## Author contributions

BP has made a substantial contribution to the concept of the article. CL revised it critically for important intellectual content. TS, WL, and AM approved the version to be published. All authors contributed to the article and approved the submitted version.
